# Identification of inhibitors that dually target the new permeability pathway and dihydroorotate dehydrogenase in the blood stage of *Plasmodium falciparum*

**DOI:** 10.1038/srep37502

**Published:** 2016-11-22

**Authors:** Benjamin K. Dickerman, Brendan Elsworth, Simon A. Cobbold, Catherine Q. Nie, Malcolm J. McConville, Brendan S. Crabb, Paul R. Gilson

**Affiliations:** 1 Burnet Institute, Melbourne, Victoria, Australia; 2 Monash University, Clayton, Victoria, Australia; 3 Bio21 Institute of Molecular Science and Biotechnology, University of Melbourne, Parkville, Victoria, Australia

## Abstract

*Plasmodium* parasites are responsible for the devastating disease malaria that affects hundreds of millions of people each year. Blood stage parasites establish new permeability pathways (NPPs) in infected red blood cell membranes to facilitate the uptake of nutrients and removal of parasite waste products. Pharmacological inhibition of the NPPs is expected to lead to nutrient starvation and accumulation of toxic metabolites resulting in parasite death. Here, we have screened a curated library of antimalarial compounds, the MMV Malaria Box, identifying two compounds that inhibit NPP function. Unexpectedly, metabolic profiling suggested that both compounds also inhibit dihydroorotate dehydrogense (DHODH), which is required for pyrimidine synthesis and is a validated drug target in its own right. Expression of yeast DHODH, which bypasses the need for the parasite DHODH, increased parasite resistance to these compounds. These studies identify two potential candidates for therapeutic development that simultaneously target two essential pathways in *Plasmodium*, NPP and DHODH.

More than 214 million cases of malaria were reported worldwide in 2015 which tragically resulted in 438,000 deaths, mainly in Africa^1^. Malaria is caused by five species of mosquito-borne *Plasmodium* parasites. Globally, *Plasmodium falciparum* causes the most morbidity and mortality, followed by *P. vivax*, which is prevalent outside of Africa. In the absence of an effective vaccine, anti-malarial drugs represent a crucial control measure with artemisinin-based combination therapies (ACTs) as the frontline drugs of choice. Worryingly, a degree of resistance to ACTs has been detected within the Greater Mekong subregion of Asia and there are serious concerns that resistance to these drugs may spread and/or emerge in other endemic regions^2^. There is an imperative to identify new drug targets in these parasites and next-generation antimalarials in order to bolster existing treatments such as ACT as well as provide new therapeutic approaches to address ongoing complications of drug-resistance.

Asexual blood stages of *Plasmodium* grow rapidly in infected erythrocytes and need to acquire a range of essential nutrients from their host cell^3^. Although the parasite is able to source the majority of amino acids through digestion of haemoglobin, isoleucine is not present in haemoglobin and must be obtained from plasma^4^. In addition, parasites require purine precursors and pantothenate from the plasma^5^,^6^. While many of these metabolites can be transported across the erythrocyte plasma membrane and be subsequently utilized by intracellular parasite stages, others are either not transported or transported at a rate that is insufficient to sustain rapid parasite growth^5^,^7^,^8^,^9^,^10^. The malaria parasite overcomes this problem by remodelling nutrient transport pathways in the erythrocyte membrane through the expression of the new permeability pathways (NPPs), which allow entry of a wide range of nutrients required for parasite growth, allowing faster permeation of smaller, anionic and non-polar solutes^11^,^12^. NPPs may also facilitate the removal of parasite metabolic waste products, such as lactate, but this has not been formally demonstrated^6^.

The NPPs are attractive drug targets, as chemically blocking them appears to arrest parasite growth and cause parasite death. High-throughput screening (HTS) of 70,000 compounds was previously performed to identify potent NPP inhibitors by measuring the sensitivity of infected erythrocytes to sorbitol mediated lysis^13^. Sorbitol enters the infected erythrocytes via the NPPs, leading to osmotic lysis of the erythrocytes, release of haemoglobin and a decrease in the turbidity of the cell suspension, which can be readily measured. While this approach identified several compounds that potently blocked parasite NPPs with K_0.5_ (drug concentration at which sorbitol uptake is inhibited by 50%) of less than 100 nM^13^, a three day parasite proliferation assay revealed the EC_50_ for growth were many fold higher at 2–15 μM. The relative lack of growth inhibition compared to the degree of NPP inhibition called into question the suitability of NPPs as drug targets. It should be noted however that these assays were performed in complex tissue culture media containing much higher levels of essential nutrients than in human serum. In human serum or in artificial media containing key nutrients at levels comparable to those in human serum, the NPP inhibitors were much more potent with EC_50_ falling to sub-micromolar levels^3^.

HTS of large compound libraries have identified many potent inhibitors of asexual blood stage parasites with relatively low toxicity to cultured human cells^14^. The challenge is to now discover the molecular targets of these drugs as a means of selecting which compounds to progress towards clinical development. To assist in this process, 200 drug-like and 200 probe-like compounds known as the Malaria Box were made freely available by the Medicines for Malaria Venture (MMV) to the research community^15^. In an effort at targeting our search for novel NPP inhibitors that could be developed therapeutically, we decided to screen the Malaria Box whose compounds have already been validated for inhibiting parasite growth with an EC_50_ < 4 μM. In place of a turbidity assay to measure sorbitol induced lysis, we used engineered parasites that export an enhanced form of luciferase, Nanoluciferase (NLuc), into their host erythrocyte cytoplasm^16^. Sorbitol-lysis and subsequent release of NLuc into media containing the enzyme’s substrate produces luminescence that is proportional to NPP activity. Here we identify two potent NPP inhibitors from the Malaria Box which were highly inhibitory to parasite growth, even in conventional culture media. Unexpectedly, further experiments revealed that in addition to inhibiting NPP, both compounds also inhibited the essential mitochondrial enzyme, dihydroorotate dehydrogenase (DHODH), which is indirectly targeted by another front line antimalarial, atovaquone. These compounds represent strong candidates for drug development as pleiotropic inhibitory effects are expected to hinder development of drug resistance.

## Results

### Measuring NPP inhibition using a luciferase reporter

**Figure 1:**
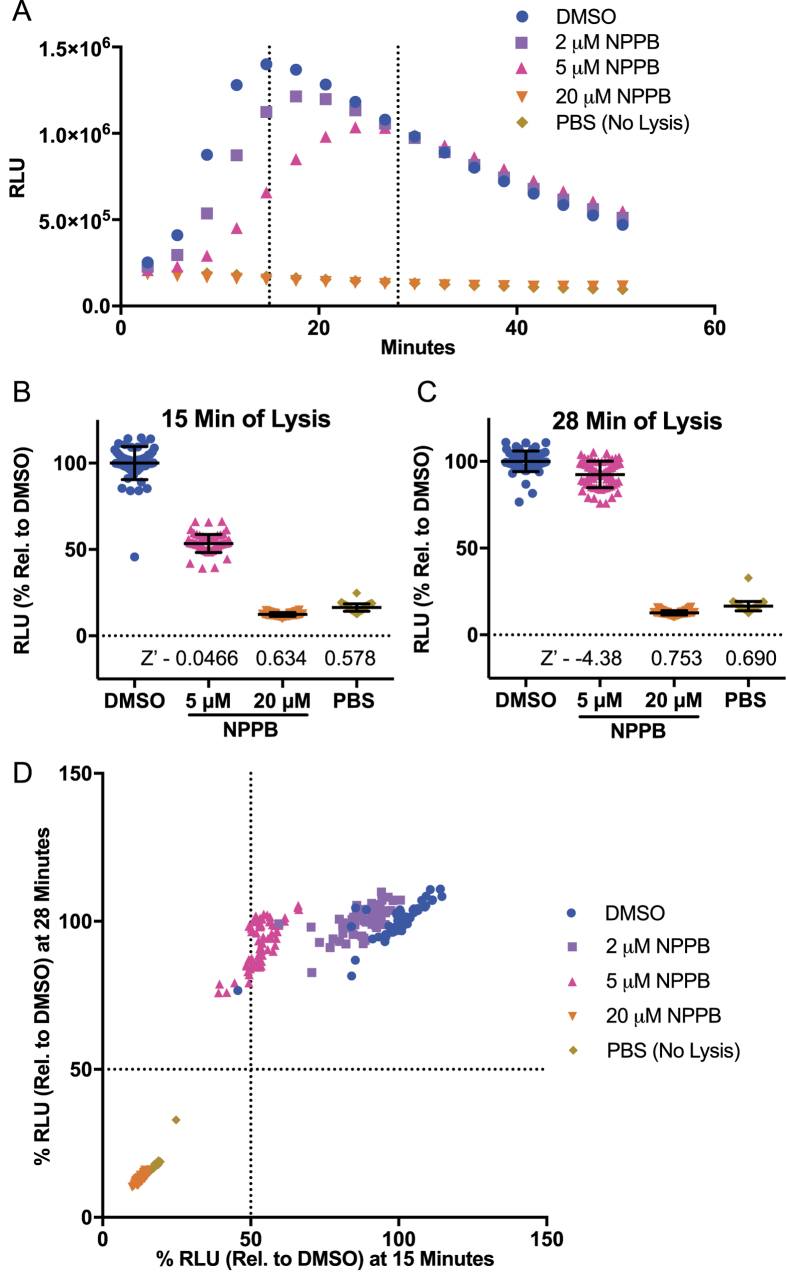
Establishment and validation of a Nanoluciferase (NLuc)-based high-throughput screening method of NPP inhibition. (**A**) Representative kinetic data from screening method. RBCs infected (iRBC) with Nluc expressing parasites were treated with NPPB prior to adding sorbitol to inhibit iRBC lysis and release of NLuc. DMSO vehicle (100% lysis) and PBS (sorbitol replacement, no lysis) controls are also shown. Dashed lines denote 15 and 28 minutes post sorbitol addition. (**B** and **C**) Relative light units (RLU) as a percentage relative to DMSO average at 15 (**B**) and 28 (**C**) minutes after addition of sorbitol for each drug treatment. Z′ values for each treatment are listed above the x-axis. (**D**) Graph of %RLU of NPPB and control-treated iRBC at 15 minutes versus 28 minutes post sorbitol addition.

We have previously shown that an exported NLuc fusion protein containing the first 115 amino acids (including the PEXEL export motif) of the *P. falciparum* exported protein Hyp-1, was exported into the iRBC cytoplasm^16^. To measure the inhibition of NPPs, erythrocytes infected with trophozoite stage parasites expressing this fusion protein (~28 hours post invasion, hpi) were treated with the general anion channel inhibitor 5-Nitro-2-(3-phenylpropylamino) benzoic acid (NPPB), which has been shown to inhibit NPPs^12^. Parasites were then diluted 5-fold in sorbitol buffer to induce lysis and subsequent release of NLuc into the sorbitol buffer. Previously, NLuc release was determined by removing unlysed cells by centrifugation and measuring luminescence in the supernatant. We have refined this approach for high throughput screening (HTS) by adding the Nano-Glo substrate directly to the sorbitol buffer, allowing the quantification of the kinetics of lysis in whole cells without requiring any further handling of the sample (Fig. 1A).

The addition of sorbitol buffer containing the Nano-Glo substrate to iRBCs resulted in a rapid, exponential increase in RLU, followed by a plateau and signal depletion as the substrate was exhausted (Fig. 1A). In contrast, addition of an equal volume of PBS containing Nano-Glo to iRBCs (no-lysis control) showed very low background signal which slowly degraded, presumably reflecting substrate instability. We then validated this approach as a high throughput method for screening inhibitors of the NPPs across a concentration range of NPPB (Fig. 1A). While 2 and 5 μM NPPB resulted in partial inhibition of lysis, 20 μM NPPB completely inhibited lysis, resulting in kinetics of NLuc release that were virtually identical to the PBS no-lysis control. As a measure of suitability for a high throughput screen (HTS) we tested the variance amongst wells using the two concentrations of NPPB to evaluate the assay for identifying a weak and a strong inhibitor of lysis. This control assay was repeated in three independent experiments on separate days, with each microplate containing 16 wells for each NPPB concentration along with DMSO and PBS no-lysis controls. This dataset was used to calculate Z′ values, a measure of the suitability of the assay for HTS based on both the dynamic range and variation of the signal with Z′ values of ≥0.5 being considered suitable for HTS^17^. We normalized luminescence (RLU) as a percentage relative to DMSO controls as 100% at 15 minutes (as an early time point during exponential increase of signal), and 28 minutes (as a late time point after plateau) (Fig. 1B,C, respectively). These data show strong statistical differences from DMSO (with p < 0.0001 calculated using unpaired, two-tailed Student’s t-test with Welch’s correction) for both concentrations of NPPB and PBS as a negative control. These data were also used to calculate Z′ values of 0.0466, 0.634 and 0.578 for 5 μM NPPB, 20 μM NPPB and no lysis controls respectively at 15 minutes (Fig. 1B), and −4.38, 0.753 and 0.690 for 5 μM NPPB, 20 μM NPPB and no lysis controls, respectively, at 28 min. (Fig. 1C). These scores demonstrate that this assay is suitable for HTS of strong inhibitors of lysis at both time points and may provide indications of weak inhibitors at 15 min. From these data, we chose to analyze 15 min and 28 min normalized RLU (% Relative to DMSO) as measures of inhibition when screening the 400 compounds from the MMV Malaria Box. As an example, 2 μM, 5 μM and 20 μM NPPB data has been similarly presented by plotting percent lysis at 15 min vs. percent lysis at 28 min (Fig. 1D).

### The initial screening of the Malaria Box revealed two potent NPP inhibitors

**Figure 2:**
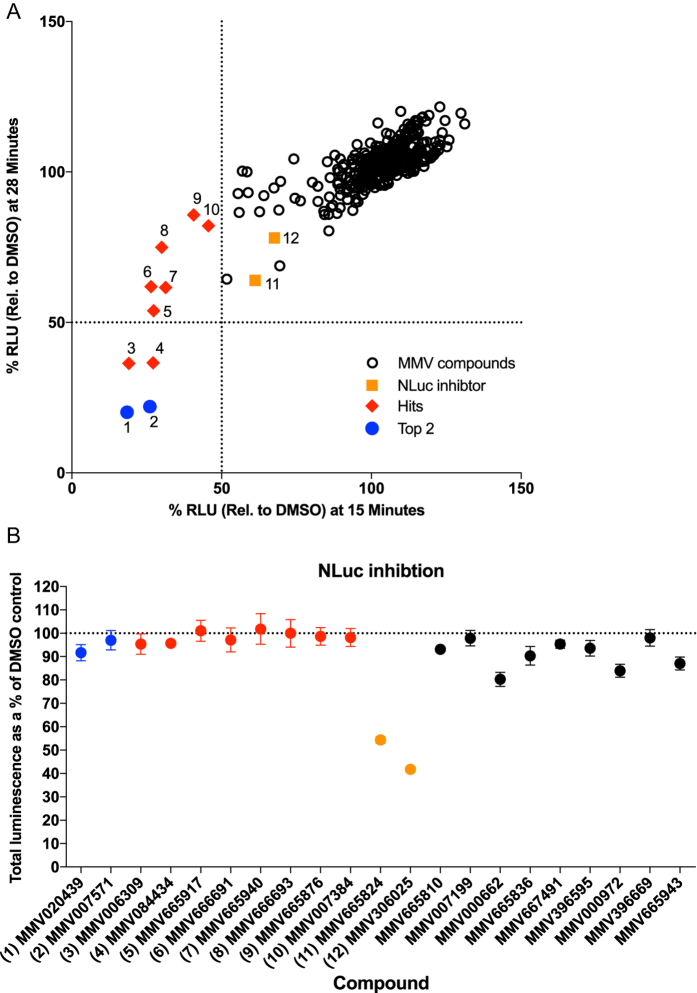
Sorbitol induced lysis of *Plasmodium falciparum* parasites expressing Nanoluciferase (NLuc) following pre-treatment with the 400 MMV Malaria Box compounds. (**A**) Inhibition of lysis by MMV Malaria Box compounds was quantified by the activity of NLuc released following sorbitol lysis and was expressed as relative light units (RLU) relative to DMSO control. (**B**) Counter screen of top 20 NPP inhibitors from the MMV Malaria Box. Inhibition of NLuc was measured by adding the MMV compounds to parasites that had been lysed in water. Two compounds MMV665824 and MMV306025 strongly inhibited NLuc activity. Colours and numbers in (**A**) correspond to those in (**B**).

We used these optimised conditions to screen all 400 compounds from the MMV Malaria Box^15^ at a final concentration of 2 μM. Using a cut-off of 50% inhibition relative to DMSO (0% inhibition) control wells, 10 compounds inhibited at 15 min, 4 of which also inhibited at 28 min (Fig. 2A). The two compounds that most strongly inhibited NPP function, MMV007571 and MMV020439, are further characterized below. The complete data set from the screen is shown in Supplementary Table S1.

To ensure that the most inhibitory compounds were not producing a false positive signal by directly inhibiting NLuc activity, we performed a counter screen on the 20 most potent compounds. To determine if any of the compounds directly inhibited NLuc activity, the iRBC were first lysed in water to release the NLuc prior to adding the MMV drugs and Nano-Glo substrate. Relative to a DMSO control, two compounds (MMV665824 and MMV306025) strongly inhibited the activity of NLuc and so were rejected for future analysis (Fig. 2B). Importantly, the two compounds that we identified as the most potent inhibitors of sorbitol-induced lysis did not inhibit NLuc (Fig. 2B).

### Characterisation of MMV020439 and MMV007571 NPP inhibition

**Figure 3:**
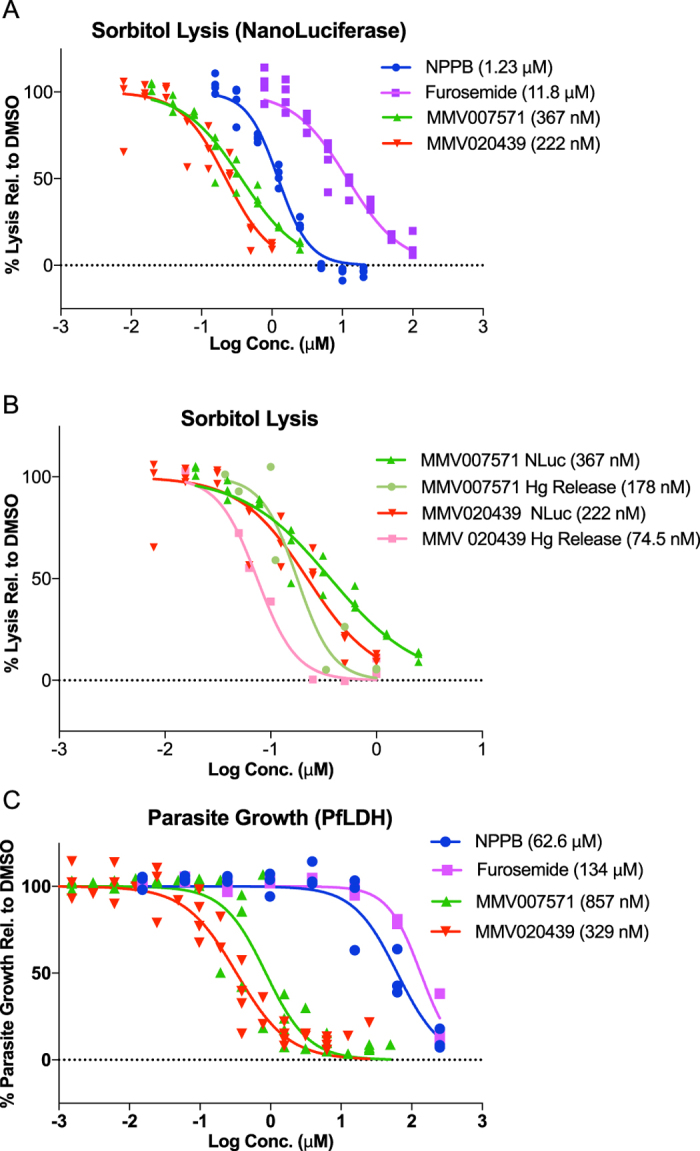
EC_50_ determination for inhibition of sorbitol lysis and parasite growth. (**A**) EC_50_ curves of sorbitol lysis of Nanoluciferase (NLuc) expressing parasites following treatment with serially diluted NPPB, furosemide and the MMV compounds. Sorbitol lysis was detected as an exponential growth rate in RLU over time (RLU/min^−1^) as a percentage between DMSO (100%) and PBS in place of sorbitol (0%). (**B**) Comparison of EC_50_ of sorbitol-induced lysis measured by NLuc activity versus haemoglobin release of MMV007571 and MMV020439 treated iRBC. (**C**) Parasite proliferation after 72 h treatment with NPP inhibitors as measured by *P. falciparum* lactate dehydrogenase (*Pf*LDH) assay. *Pf*LDH levels were measured at absorbance 620 nm as a percentage between DMSO (100%) and uninfected RBCs (0%) to determine EC_50_ for parasite proliferation. In all graphs, values in parentheses indicate EC_50_ for each drug.

MMV020439 and MMV007571 were independently sourced from MolPort and further tested for inhibition of lysis at various concentrations. Rather than continuing to evaluate luminescence at two time points, we refined the analysis of our assay to include more of the kinetic data by fitting the exponentially increasing portion of the data to an exponential growth equation to derive the rate of increase (see materials and methods for details). This allowed us to evaluate a single value that takes both early and late signal into account. We reanalysed our initial validation data (from Fig. 1B and 1C) to calculate Z′ values for this modified analysis, resulting in 0.104 for 5 μM NPPB, 0.642 for 20 μM NPPB and 0.711 for PBS as a no-lysis control demonstrating the validity of this analysis (Fig. S1). The percentage lysis, as calculated by exponential growth rate relative to DMSO controls (as 100%) and PBS no-lysis controls (as 0%) were plotted against drug concentration to determine the EC_50_ for inhibition of sorbitol lysis, demonstrating sub-micromolar inhibition of NPP-mediated lysis by both compounds (Fig. 3A and Table 1). Both of the MMV compounds were several-fold more potent (367 nM and 222 nM for MMV007571 and MMV 020439 respectively) than the control NPP inhibiting compounds, NPPB (1.23 μM) and furosemide (11.8 μM)^12^.

To independently confirm the compounds were inhibiting the NPP we measured haemoglobin release from sorbitol-lysed iRBC as a marker for NPP inhibition. The EC_50_’s calculated using both assays were similar with both compounds inhibiting slightly more strongly in this assay compared to our NLuc assay (Fig. 3B).

### The MMV NPP inhibitors are also potent growth inhibitors of *P. falciparum* proliferation

We further characterized the NPP inhibitors by examining their parasiticidal activity. *P. falciparum* 3D7 strain iRBCs were incubated with various concentrations of each compound, along with the previously described control compounds NPPB and furosemide, to determine their ability to inhibit parasite growth. Using a colorimetric assay of *P. falciparum* lactate dehydrogenase (PfLDH) activity, we determined the percentage parasite proliferation relative to DMSO controls as 100% and uninfected RBCs as 0% following 72 h of culture (Fig. 3C). Interestingly, NPPB required ~50-fold higher concentration for 50% inhibition of parasite growth compared to NPP inhibition (62.6 μM), while furosemide required ~11-fold higher concentrations (134 μM), and most importantly, MMV007571 and MMV020439 required ~2 and ~1.5-fold higher concentrations (857 nM and 329 nM), respectively. This may be in part due to NPPB and to some extent furosemide losing their activity in the presence of human serum components, as the sorbitol lysis assay was done in PBS for a total of 1 hour and 20 minutes, while the parasite growth assay was done in serum containing media over 72 h. It is important to point out that while NPPB and furosemide partially lose their inhibition of sorbitol lysis when the assay is done in the presence of serum, both MMV007571 and MMV020439 remain active (Fig. S2). As any therapeutic inhibitors of NPP will need to function in the presence of human serum, this further validates the potential utility of these two compounds for future antimalarial development.

### Metabolite profiling of NPP inhibitors indicates they also block *Pf*DHODH

**Figure 4:**
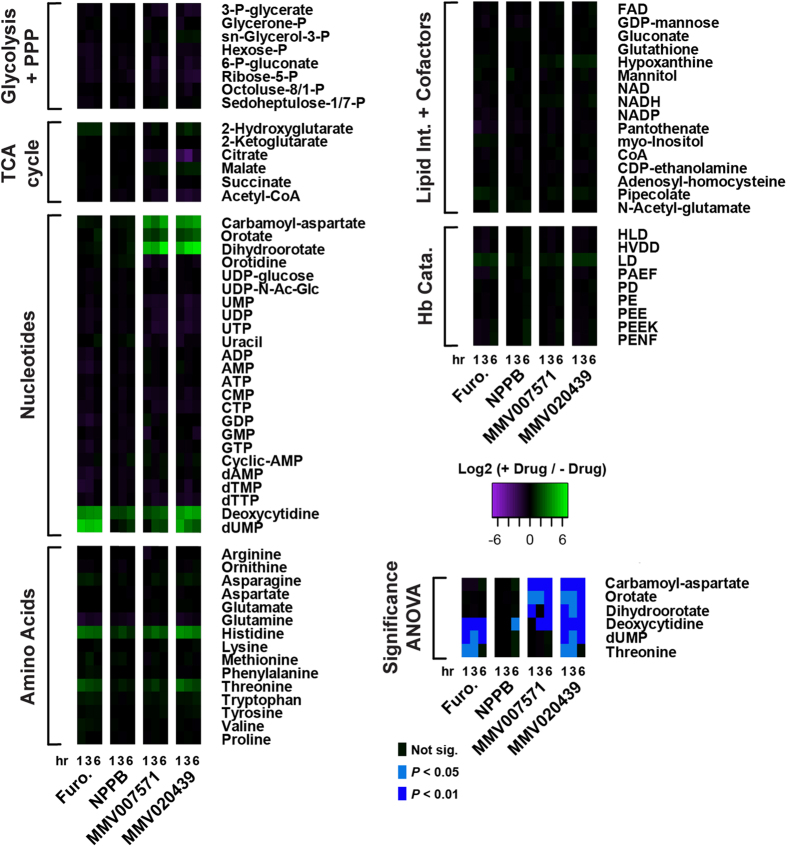
Metabolite profiles of *P. falciparum*-infected erythrocytes following treatment with NPP inhibitors. Metabolite pools (measured as ion counts) collected following 1, 3 or 6 hours of drug treatment were detected via LC-MS and compared to untreated controls. Changes in total metabolite pools are expressed as the log_2_ ratio of treated (+drug) to untreated controls (−drug). The data are presented as the mean of 3–5 independent experiments. Drug treatments are as follows: Furosemide (Furo.; 200 μM), NPPB (50 μM), MMV007571 (5 μM) and MMV020439 (5 μM). Statistical significance was determined using a one-way ANOVA with Tukey post-hoc testing. Statistical significance is indicated on the heatmap (with ‘Not sig.’ indicating a post-hoc *p*-value > 0.05).

To further investigate the mode of action of these two putative NPP inhibitors, we analysed the metabolic phenotype in *P. falciparum*-infected erythrocytes treated with MMV020439 or MMV007571. Previous work has characterized the metabolic phenotypes associated with several antimalarials and known inhibitors; the inhibitory profile of NPP inhibitors however has not been tested^18^. It was therefore necessary to first determine the metabolic perturbations induced following NPP inhibition by the control compounds NPPB and furosemide. Complete metabolic profiling data has been provided as Supplementary Table S2. Furosemide treatment resulted in a significant accumulation of threonine, deoxycytidine and dUMP (Fig. 4; 1.59 ± 0.10, 4.02 ± 0.16, and 5.43 ± 0.28; log_2_ fold-change; Mean ± SEM) over six hours. These changes appear to be specific, as indicated by the lack of change in other metabolites detected, and matched the profile during NPPB exposure (albeit only significant for deoxycytidine after 6 hours).

**Figure 5:**
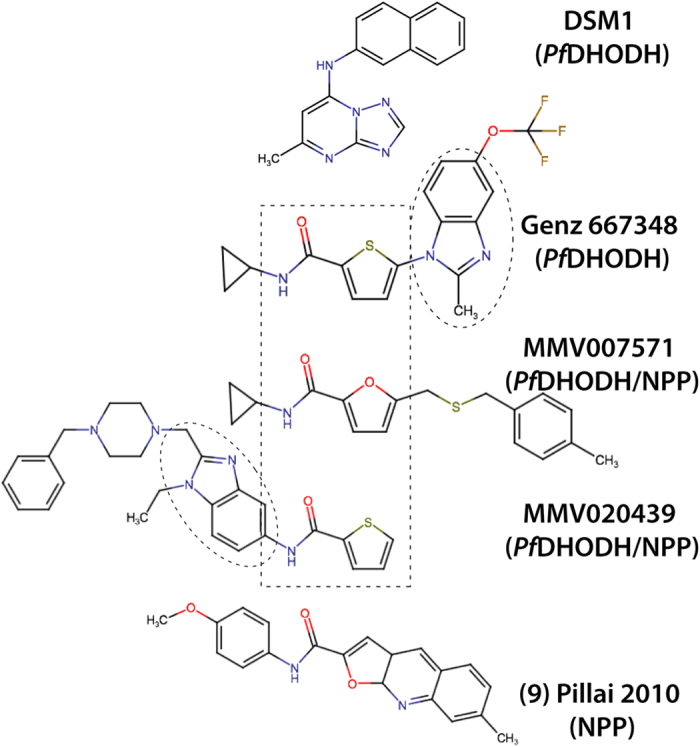
Structures of known *Pf*DHODH inhibitors and NPP inhibitors. Structures of known *Pf*DHODH inhibitors Genz-667348^21^ and DSM1^19^ compared to NPP inhibitors MMV007571, MMV020439 and compound 9^13^.

Treatment with MMV007571 or MMV020439 elicited the same metabolic phenotype as the NPP inhibitors (accumulation of threonine, deoxycytidine and dUMP) but also induced a secondary phenotype (Fig. 4). Exposure with either MMV020439 or MMV007571 resulted in a significant increase in the intracellular N-carbamoyl-aspartate (4.43 ± 0.26 and 4.49 ± 0.22; log_2_ fold-change; Mean ± SEM, after 6 h, respectively), dihydroorotate (6.56 ± 0.56 and 6.54 ± 0.53; log_2_ fold-change; Mean ± SEM, after 6 h, respectively), and orotate pools (2.29 ± 0.18 and 2.58 ± 0.36; log_2_ fold-change; Mean ± SEM, after 6 h, respectively). This phenotype is consistent with inhibition of *Pf*DHODH, which converts dihydroorotate to orotate, resulting in a build-up of upstream intermediates^18^. These findings confirm treatment with either MMV020439 or MMV007571 induces a metabolic phenotype consistent with inhibition of NPP, but also suggests that the two compounds inhibit *Pf*DHODH either directly (as with the compound DSM1^19^) or indirectly (as with *bc*_1_ complex inhibition by atovaquone^20^) as well. Upon closer inspection of the structure of MMV007571 and to a lesser extent MMV020439 we found similarities to the benzimidazole *Pf*DHODH inhibitor Genz-667348 21, but not triazolopyrimidine inhibitors such as DSM1 and DSM265 (Fig. 5)^22^,^23^, further supporting our hypothesis that these compounds target *Pf*DHODH as well as NPP function.

### Complementation with yeast DHODH partly rescues *P. falciparum* from the inhibitory *Pf*DHODH effects of the NPP inhibitors

Our metabolomics data suggested that both MMV compounds have dual activity against DHODH and NPP. It is possible that these pathways interact, and that we are seeing multiple effects from a single mechanism of inhibition. To investigate this further, we generated parasites expressing *Saccharomyces cerevisiae* DHODH (yDHODH), which shares little homology with *Pf*DHODH and is not dependent on the cofactor ubiquinone. This line was further transfected with Hyp1-Nluc to allow measurement of NPP activity. We first tested the yDHODH parasites with the control compounds NPPB and furosemide and found that compared to the parental 3D7 parasites, proliferation was unaffected by expression of yDHODH (Fig. S3A,B). To ensure that the yDHODH was functional, parasites were exposed to the mitochondrial respiratory chain and *Pf*DHODH inhibitors, atovaquone (Atv) and DSM1^24^, respectively, and shown to be highly resistant to both inhibitors (Fig. S3C,D). We then tested the sensitivity of the yDHODH parasites to MMV007571 and MMV020439 using the *Pf*LDH proliferation assay and found there was a partial effect in which the EC_50_ of MMV007571 was shifted to an approximately 40-fold higher concentration, while there was a more modest effect on MMV020439, shifting the EC_50_ to about a 6-fold higher concentration (Fig. S4A,C, Table 2). Importantly, sensitivity to sorbitol lysis was unchanged (Fig. S4B,D). These results indicate that both compounds are inhibiting *Pf*DHODH, although this represents only part of their activity. Furthermore, cells expressing yDHODH are more resistant to MMV007571 than they are to MMV020439 indicating that MMV007571 elicits more of its activity through inhibition of *Pf*DHODH than that of MMV020439. Importantly, this result suggests that inhibition of NPP and DHODH are independent, supporting our hypothesis that these compounds have two targets.

### MMV020439 inhibits *Pf*DHODH through cytochrome *bc1* while MMV007571 inhibits both enzymes

Inhibition of *Pf*DHODH can be achieved directly or indirectly by inhibition of cytochrome *bc1* and the recycling of the cofactor ubiquinone. It is well established that proguanil (whose metabolised form cycloguanil, inhibits dihydrofolate reductase) synergises with cytochrome *bc1* inhibitors both increasing their potency and abrogating yDHODH-conferred resistance^25^,^26^. To determine whether our MMV compounds were inhibiting *Pf*DHODH directly or indirectly by targeting *bc1* all the inhibitors were reanalysed in the presence or absence of 1 μM proguanil and the results are summarised in Tables 3 and 4. Beginning with atovaquone, we observed the EC_50_ decrease by ~11-fold in the presence of 1 μM proguanil in 3D7 (Fig. S5A). This shift was not observed using the direct inhibitor of *Pf*DHODH DSM-1 in 3D7, demonstrating synergy between atovaquone and proguanil (Fig. S5B). Expression of yDHODH, as in previous experiments, resulted in a high degree of resistance to both atovaquone and DSM-1 but in the presence of proguanil, yDHODH parasites regain sensitivity (with an EC_50_ within 1 fold of 3D7 parasites) to atovaquone but remain highly resistant to DSM-1 (Tables 3 and 4). When the same experiment was performed with MMV020439 and MMV007571, yDHODH parasites regained sensitivity to both compounds in the presence of proguanil (Fig. S5C,D). Additionally, MMV020439 showed the same degree of synergy (~14-fold decrease in EC_50_) with proguanil, whereas MMV007571 showed only a modest increase in potency (~3.5 fold decrease in EC_50_) (Table 3). Taken together, these results suggest that both compounds inhibit *Pf*DHODH indirectly through inhibition of cytochrome *bc1*.

In an alternate approach, we sought to test inhibition of *Pf*DHODH directly using an *in vitro* enzymatic assay. Recombinant *Pf*DHODH was purified and activity measured as previously described^27^, summarised in Table 5. Even at extremely high concentrations of atovaquone (100 μM, ~170,000x EC_50_ for parasite growth) there was no significant inhibition of *Pf*DHODH activity (Fig. S6A,E). In contrast, DSM-1 inhibited DHODH *in vitro* with an IC_50_ of 441 nM, ~4.5x EC_50_ for parasite growth (Fig. S6B,E). MMV020439, like atovaquone, failed to inhibit *Pf*DHODH *in vitro* at the highest concentration tested, 250 μM (~800 fold EC_50_ for parasite growth), supporting our proguanil results that suggest that this compound indirectly inhibits *Pf*DHODH through cytochrome *bc1* (Fig. S6C,E). MMV007571 however inhibited DHODH *in vitro* with an IC_50_ of 158 μM, ~144x EC_50_ for parasite growth (Fig. S6D,E). Together with our experiments using proguanil, these results indicate that MMV007571 inhibits *Pf*DHODH directly as well as indirectly through cytochrome *bc1* (Table 5).

### MMV020439 potency is increased under reduced nutrient conditions

Previous attempts to identify NPP inhibitors have demonstrated that potent inhibitors of NPP activity do not correlate with strong inhibition of parasite growth^13^. This may reflect residual nutrient uptake when parasites are cultivated in nutrient-rich medium *in vitro*. Pillai *et al*. therefore developed a media that more closely resembles human serum and contains reduced concentrations of isoleucine, glutamine and hypoxanthine (PGIM media), all of which are imported through NPPs^3^. To confirm that reduced NPP function caused by our MMV compounds was also contributing to parasite growth inhibition along with the *Pf*DHODH mechanism we tested whether our MMV compounds would inhibit parasite growth more effectively in PGIM media. For this experiment, two predictions were made: (1) the EC_50_ for parasite growth should decrease as the compounds become more effective in PGIM, and (2) as inhibition of NPP contributes more to the overall parasiticidal effect of these compounds in PGIM, resistance conferred by yDHODH expression should be less dramatic. The results of this experiment were that we observed a decrease in EC_50_ for both NPPB and furosemide, although only furosemide was statistically significant (3.3 fold reduction in EC_50_, p = 0.0216) (Tables 6 and 7). For 3D7 MMV020439 had a dramatic reduction (5.7 fold, p = 0.0304) in EC_50_ in PGIM media compared to normal media, as well as almost completely abrogating the yDHODH-conferred resistance (from 52 fold (p = 0.0023) in normal media to 4.3 fold [ns]) in PGIM (Tables 6 and 7). Although there was no significant decrease for 3D7 in EC_50_ for MMV007571, yDHODH-conferred resistance was reduced from 17-fold (p = 0.001) in normal media to 6.9 fold (p = 0.0321) in PGIM (Tables 6 and 7).

## Discussion

To identify more potent NPP inhibitors we took advantage of the Malaria Box library which contains 400 compounds derived from several HTS that have collectively identified >20,000 compounds that were highly inhibitory to parasite growth^14^,^28^,^29^. Chemical inhibition of iRBC lysis has traditionally been expressed as a K_0.5_ value which is the drug concentration at which sorbitol uptake is inhibited by 50% 13. Here we used a luciferase based screening method that required fewer handling steps than traditional methods to directly measure lysis in whole parasite cultures containing fewer than 5% iRBCs^13^,^30^,^31^. This approach allowed us to derive an EC_50_ for inhibition of sorbitol lysis, which for furosemide was 11.76 μM compared with the previously reported K_0.5_ for this compound of 2.7 μM^32^. Screening of the Malaria Box revealed two potent NPP inhibitors, MMV020439 and MMV007571. The EC_50_ values for MMV020439 and MMV007571, of 222 and 367 nM respectively, are in a similar range as the K_0.5_ of the best NPP inhibitors previously identified from a HTS^13^. Interestingly, the EC_50_ for parasite growth of our Malaria Box inhibitors indicated they were several-fold more potent than those identified previously^13^ which is not wholly unexpected given our approach of screening compounds previously identified as potent inhibitors of parasite growth^15^.

To confirm that the two Malaria Box NPP inhibitors had the same global effects on parasite metabolism as other NPP inhibitors we performed an untargeted metabolomic analyses of drug treated parasites. Treatment with furosemide or NPPB induced a specific metabolic signature that was distinct from that produced by a variety of other anti-malarial compounds^18^. The mechanisms underlying this inhibitory metabolite profile are uncertain given the complexity in interpreting steady-state metabolite abundance. Several phenomena could be contributing to the observed metabolic phenotype, including: altered substrate distribution across multiple compartments, redundancy of transporters for many NPP substrates, multiple metabolic pathways converging on NPP substrates, and altered metabolic turnover of pathways associated with NPP substrates. Nutrient import aside, NPPs are probably also required for the elimination of waste products from the digestion of hemoglobin, since only a fraction of the amino acids are utilized and NPP blockage could elevate certain abundant amino acids^33^. We anticipated that a signature of NPP blockage would be a deficit of isoleucine since this needs to be imported. Unfortunately however, isoleucine and leucine could not be adequately separated chromatographically and we therefore could not easily discriminate between these so they were not included in the analysis. Notwithstanding, this work demonstrates that metabolite profiling can identify phenotypes associated with membrane transporter inhibition. However, the classical ‘accumulation upstream/decrease downstream’ observed for inhibition of metabolic enzymes, may not be applicable for inhibition of transporters (particularly those with a diverse substrate range) because of the reasons listed above.

The metabolic phenotype generated by MMV020439 and MMV007571 treatment closely matched this NPP inhibition profile, further validating that the compounds were blocking NPPs. However, both Malaria Box compounds also induced a number of additional changes in metabolite levels (compared to NPPB and furosemide) including the accumulation of carbamoyl-aspartate, dihydroorotate and orotate. These metabolites are intermediates in the essential and non-redundant pathway of pyrimidine synthesis in the parasite. The rate-limiting step in pyrimidine biosynthesis is the oxidation of dihydroorotate to orotate by the enzyme *Pf*DHODH, which is a promising drug target with the inhibitor DSM265 under clinical development^34^.

Ectopic expression of yDHODH conferred 6- & 40-fold increases in the EC_50_ for growth against MMV020439 and MMV007571, respectively, suggesting that both compounds particularly MMV007571, target *Pf*DHODH either directly or indirectly. Interestingly, while the reversal of yDHODH resistance to both MMV compounds in the presence of proguanil suggested that cytochrome *bc1* is the target, MMV007571 also inhibited recombinant *Pf*DHODH *in vitro*, suggesting complex direct and indirect modes of action. The similarity of the MMV compounds, particularly MMV007571, to Genz-667348 suggested they might function similarly. As both *Pf*DHODH and cytochrome *bc1* bind ubiquinone, and most *Pf*DHODH inhibitors including Genz-667348 function by competing with ubiquinone for occupation of the enzyme’s hydrophobic cleft^21^, it is possible that a small molecule such as MMV007571 could inhibit both enzymes through a common mechanism.

A recent study where hundreds of assays were used to interrogate the Malaria Box for mechanisms of action^35^ used a similar yDHODH/proguanil reversal strategy to establish that 15 of 400 compounds target *Pf*DHODH and 11 compounds target the *bc1* complex. Interestingly though, it was reported that the resistance of yDHODH parasites to MMV020439 and MMV007571 only improved 3-fold over the parental line Dd2, and so they were not identified as *Pf*DHODH inhibitors. It is important to mention however that this report did show reversal of yDHODH conferred resistance with proguanil, consistent with our report. We found that yDHODH conferred much greater resistance to both MMV compounds, possibly due to the different parasite lines used (3D7 cf Dd2) and the yDHODH expression systems. It is also possible that the parasite lines were differentially susceptible to NPP inhibition because of polymorphisms of *clag 3.1* genes, which are thought to play a role in NPP function^36^.

It is unknown at this stage if the similarity between the structures of MMV007571 and MMV020439 mean they are likely to bind to the same NPP protein. We compared the structures of the Malaria Box compounds to NPPB, furosemide and other compounds identified from HTS for NPP inhibitors such as ISPA-28^3^,^13^,^36^. The only obvious similarity was to compound **9**, one of the most potent hits identified^13^. This weak similarity of compound **9** to MMV007571 and MMV020439 may provide some insight into the mechanism of inhibition, although this will need to be studied in more detail.

In summary, we have identified two potent NPP inhibitors, MMV007571 and MMV020439, from the Malaria Box library of highly growth inhibitory compounds. Metabolomic analysis suggested that these two compounds also target the *P. falciparum* DHODH enzyme. Importantly, the incomplete resistance conferred by yDHODH under normal conditions, and the increased potency (MMV020439) and decreased resistance conferred by yDHODH (both MMV compounds) in PGIM media demonstrate that both targets contribute to the inhibition of parasite growth. As drug resistance has and continues to be a major problem for the control and elimination of malaria, compounds that have multiple targets such as those we have identified here could be important leads for future therapeutic development since it would be anticipated that parasites would take longer to develop resistance.

## Methods

### Parasites

*Plasmodium falciparum* 3D7 parasites were cultured as per^37^ in RPMI media supplemented with 25 mM HEPES, 25 mM Sodium Bicarbonate, 367 μM Hypoxanthine, 31.25 μg/mL Gentamicin, 5% Heat-inactivated human serum (Australian Red Cross) and 0.25% Albumax II (Invitrogen). To generate parasites expressing exported-NLuc, erythrocytes electroporated with 100 μg of plasmid DNA encoding the first 115 aa of the PEXEL-containing protein Hyp1 fused to Nanoluciferase (Hyp1-NLuc) were infected with trophozoite stage parasites^16^,^38^,^39^. The parasites were then cultured in 2.5 nM WR99210 to establish a transfected population, and then continuously to maintain expression. The DHODH coding sequence from *Saccharomyces cerevisiae* was amplified from the plasmid pUF1-Cas9 40 with the primers DHODH_F (ATATCAGCTCGAGGTCCCATGGCAGCCAGTTTAACT) and DHODH_R (TTAAATCTGCAGTTAAATGCTGTTCAACTTCCCA), digested with XhoI and PstI and ligated into the hDHFR selectable pEF-Luc-GFP-HA^41^ digested with the same enzymes. This plasmid was then electroporated into erythrocytes which were infected with *P. falciparum* 3D7 parasites and selected with 2.5 nM WR99210 as described above. Two independent transfections were performed to establish independent lines, which were then subsequently used to infect erythrocytes electroporated with Hyp-1-NLuc in a modified pEF vector expressing blasticidin-S deaminase. These parasites were then selected with 2 μg/mL blasticidin-S (in addition to 2.5 nM WR99210) to first establish a stably expressing pool and then continuously to maintain expression.

### Compounds

Unless stated otherwise all compounds were dissolved in DSMO. 5-Nitro-2-(3-phenylpropylamino)benzoic acid (NPPB) and Furosemide were purchased from Sigma-Aldrich. Atovaquone was purchased from AK Scientific, DSM-1 was a kind gift from Susan Charman. Supplementary MMV Malaria Box compounds were obtained from MolPort (MMV007571 Molport catalog number is 007-936-716, MMV020439 Molport catalog number is 010-755-382). MMV compound SMILES are shown with NPP screening data in Supplementary Table S1.

### NPP lysis assay and statistical validation for HTS

Synchronous Hyp1-NLuc parasites at 28 hours post invasion (hpi) were washed in PBS and diluted to a final density of 1% haematocrit in a 96 well microplate with NPP inhibitors at various concentrations along with a DMSO vehicle control for 20 min at room temp. 10 μL of drug-treated parasites were then transferred to a Greiner Lumitrac microplate and placed in a CLARIOstar luminometer (BMG labtech). To each well, 40 μL of sorbitol buffer (280 mM sorbitol, 20 mM Na-HEPES, 0.1 mg/ml BSA, pH 7.4), containing Nano-Glo substrate (Promega, 1:1000 dilution) was injected and luminescence recorded at 3 minute intervals with the gain set to 2500. Where indicated, 40 μL PBS containing 1:1000 Nano-Glo substrate was used in place of sorbitol as a no-lysis control. The drug concentrations expressed are those after sorbitol dilution. For initial validation, RLU at 15 and 28 minutes was expressed as a percentage relative to the average RLU of DMSO controls. The mean and standard deviation of 48 total wells (16 wells per plate on three separate days) for each drug concentration were used to calculate Z′ values according to the equation^17^:


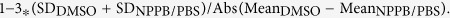


### Screening the MMV Malaria Box

The Malaria Box compounds were first diluted to a 12.5 μM solution in PBS. Whole Hyp1-NLuc parasites at the desired hpi were washed 2x times in PBS and were then mixed to 1% haematocrit with the MMV compound to give a final concentration of 10 μM. Following 20 mins incubation, lysis was induced by the addition of four volumes of sorbitol buffer containing 1:1000 Nano-Glo substrate. This resulted in a final concentration for each of the compounds at 2 μM. Luminescence upon sorbitol lysis was measured at 3 minute intervals in a luminometer for one hour. DMSO was used as a control for 100% lysis. Each microplate contained 40 compounds in duplicate as well as DMSO control wells. The level of inhibition was determined at 15 and 28 min after addition of sorbitol. Percentage inhibition was calculated relative to the mean of DMSO wells (100% lysis). For analysis of all compounds the mean inhibition of duplicate wells was used.

### Counter Screen

The ensure the compounds that produced a low RLU signal did so by reducing sorbitol lysis and not by inhibiting NLuc, the Hyp1-NLuc parasites were lysed in water to release the total NLuc. Addition of MMV drugs, sorbitol and Nano-Glo were then performed as above and luminescence recorded at a single time-point. Percentage inhibition of NLuc was calculated relative to the mean of DMSO (no inhibition) controls.

### Validation of NPP inhibitors

To calculate the EC_50_ for sorbitol lysis inhibition of the two strongest Malaria Box hits, MMV007571 and MMV020439, the rate of RLU increase per minute was calculated during the early exponential phase of lysis phase before the signal plateaued and declined due to substrate exhaustion. Data following signal plateau was excluded to maximize R^2^ (to a minimum of 3 data points) after fitting to the exponential growth equation RLU = RLU_0*_e^KT^ where T = time after lysis in minutes and K = Rate. Rate was expressed as a percentage between DMSO controls (as 100%) and uninfected RBCs (as 0%), plotted versus log [Drug (μM)], and fit to a sigmoidal dose response curve using PRISM 6 (Graphpad) to calculate EC_50_ values. To validate that this approach was reproducible, Z′ scores were calculated with the same NPPB data set used to validate single timepoint measurements.

### Haemoglobin lysis assay

This experiment was performed essentially as previously described^42^. Briefly, parasite cultures between 10–20% parasitemia approximately 34 h post-invasion were washed twice in PBS and resuspended at 50% hematocrit in PBS. Drugs (or DMSO) were diluted at 10x working concentrations in sorbitol buffer (as above without Nano-Glo substrate). 75 μL of 10x drug dilution or DMSO was added to 600 μL of soribitol lysis buffer in Eppendorf tubes (or 675 uL PBS for a no-lysis control). 75 μL of parasite culture (50% haematocrit in PBS) was added to each tube, which were incubated at 37 °C. At 2.5, 5, and 10 min, 150 μL of mixture was transferred to a fresh tube and centrifuged for 15 s at 17,000 × g. Two replicate samples of 50 μL of supernatant were collected without disturbing the cell pellet and transferred to a 96 well plate. Once all samples were collected, absorbance was measured at 540 nm to quantify haemoglobin present in the supernatant, plotted versus time (in minutes). Slope of a linear regression was used as a measure of the rate of lysis, expressed as a percentage between DMSO controls (as 100%) and uninfected RBCs (as 0%), plotted versus log [Drug (μM)], and fit to a sigmoidal dose response curve using PRISM 6 (Graphpad) to calculate EC_50_ values.

### Parasite growth assays

To determine how inhibitory the NPP inhibitors were for parasite proliferation 0.5% ring stage 3D7 parasites were cultured at 1% haematocrit in a dilution series of the NPP inhibitors. Compounds were serially diluted in DMSO at 2x working concentration and were added to cultures with a final concentration of 0.1% DMSO. Where indicated, 1 μM proguanil was included from a 100 μM stock in complete media in addition to inhibitors being tested. The growth assays were performed in triplicate for 1.5 cell cycles (37 °C, 72 h). 20 μL each well of parasite culture was then transferred to a new well containing 100 μL lysis buffer (0.1 M Tris, 222 mM lactic acid, 0.2% v/v Triton X-100 and 0.4 mg/mL acetylpyridine adenine dinucleotide [Sigma], pH 9). The reaction was initiated by adding 20 μL of a solution of 0.1 mg/ml phenozine ethosulfate [Sigma] and 2 mg/mL nitro blue tetrazolium [Sigma]. Once DMSO control wells developed a purple color, the reaction was stopped by adding 70 μL 1 M acetic acid and absorbance was measured at 620 nm. Absorbance values were expressed as a percentage between DMSO controls as 100% and uninfected RBCs as 0%, plotted versus log [Drug (μM)], and fit to a sigmoidal dose response curve using PRISM 6 (Graphpad) to calculate EC_50_ values.

### LC-MS detection of metabolite abundance

Trophozoite stage *P. falciparum*-infected cultures were enriched to >95% infected cells via magnetic enrichment. After recovery in fresh media (one hour at 37 °C) parallel time series were initiated with the addition of 200 μM furosemide, 50 μM NPPB, 5 μM MMV020439 or 5 μM MMV007571 (and DMSO vehicle treated control cultures), in complete 1640 RPMI (w 0.5% Albumax II; 0.4% haematocrit). Cultures were returned to controlled atmospheric conditions (5% CO_2_, 1% O_2_ and 94% N_2_) and incubated at 37 °C. At 1, 3, and 6 hours of drug-treatment, 1 × 10^8^ cells were pelleted, washed with 1 mL ice-cold PBS and extracted with 200 μL of 80% acetonitrile (20% water; containing 5 μM ^13^C-aspartate as an internal standard). The suspension was removed, briefly vortexed, centrifuged at 14,000 g for 10 minutes (at 4 °C) and the supernatant transferred to an MS vial. Metabolite samples were separated on a SeQuant ZIC-pHILIC column (5 μM, 150 × 4.6 mm, Millipore) using a method previously described^18^. MS detection was performed on an Agilent Q-TOF mass spectrometer 6550 operating in negative ESI mode. The scan range was 85–1700 m/z between 5 and 35 minutes at 0.9 spectra/second. Following alignment, metabolites were assigned using exact mass (<5 ppm) and retention time (compared to a standards library of 150 compounds run the same day) with the MAVEN software package^43^. The area top for each positively assigned metabolite was integrated and the log_2_ ratios (+drug/−drug) across the time series were calculated and then plotted using a heatmap script in R programming language. One-way ANOVAs were run for each time point collected using the MetaboAnalyst 3.0 package^44^. Raw ion counts were log_2_ transformed, normalized to the total ion count sum and filtered by the interquantile range with Tukey’s post-hoc test.

### Cloning, expression and purification of *Pf*DHODH

The coding sequence of *Pf*DHODH amino acids 169–569 was synthesized and codon optimised for *Escherichia coli* expression by Genscript, cloned into pProEX-HTB using NcoI and SalI restriction enzymes, and transformed into *E. coli* BL21(DE3). Cells were grown in LB containing 0.1 mM flavin mononucleotide (FMN) to an OD of 0.6. Expression was then induced by adding isopropyl β-D-1-thiogalactopyranoside (IPTG) to 1 mM and incubating cells at room temperature overnight. Cells were harvested by centrifugation, resuspended in lysis buffer (50 mM Tris, 2% Triton X100, 0.5 mM FMN, 1 mM DTT, 1x Complete protease inhibitor cocktail (Roche), 1 mg/ml lysozyme, pH 8.5), incubated at room temperature for 30 min, then frozen at −20 °C overnight. Lysate was thawed on ice and centrifuged at 20,000 × g for 30 minutes prior to binding to a 5 ml HisTrap Crude FF column (GE) equilibrated in buffer A (50 mM Tris, 300 mM NaCl, 0.1 mM FMN, 1 mM DTT, 20 mM Imidazole, 10% glycerol, pH 8.5). The column was extensively washed with buffer A and eluted in a linear gradient from buffer A to buffer B (50 mM Tris, 300 mM NaCl, 0.1 mM FMN, 1 mM DTT, 300 mM imidazole, 10% glycerol, pH 8.5). The peak fractions were pooled and dialysed against 100 mM HEPES, 150 mM NaCl, 10% glycerol, pH 8.0 and stored at −80 °C.

### *in vitro* DHODH activity assay

The assay was essentially done as in ref. 26. Briefly, each inhibitor was serially diluted in 2x activity buffer (100 mM HEPES, 150 mM NaCl, 10% Glycerol, 0.05% Triton X100, 240 μM 2,6-dichloroindophenol (DCIP), 400 μM L-dihydrorotate, pH 8.0) in a final volume of 50 μl inhibitor + buffer per well. To initiate the reaction, 50 (mu)/100nM *Pf*DHODH in 100 mM HEPES, 150 mM NaCl, 0.05% Triton X100, 10% glycerol, pH 8.0 was added to each well. Buffer without *Pf*DHODH was added to control wells. The absorbance was monitored at 620 nm every 90 seconds for 45 minutes and compared to a standard curve of DCIP to calculate the concentration of oxidised DCIP. The reduction in absorbance was essentially linear for the first 10 minutes, and so these data points were plotted against time to calculate the enzyme velocity (V, nmol/sec), which was in turn plotted as a percent relative to DMSO control and plotted against inhibitor concentration to calculate IC_50_.

### Nutrient-restricted Parasite Growth Assay

PGIM media was prepared according to ref. 3. Briefly, RPMI without amino acids (US Biologicals) was prepared as a 2x stock including 50 mM sodium carbonate and 50 mM HEPES. Amino acid “cocktails” were prepared containing all amino acids at normal RPMI concentrations, or with reduced concentrations of isoleucine (11.4 μM final concentration in 1x media compared to 381 μM in RPMI) and glutamine (102 μM final concentration in 1x media compared to 2 mM in RPMI). “Normal media” was prepared by combining the cocktail containing all amino acids at RPMI concentrations, hypoxanthine was added to a final concentration of 367 μM, heat-inactivated human serum extensively dialysed against water was added to 10%, and 2x amino acid free media was added to a final concentration of 1x. “PGIM media” was prepared by combining the cocktail containing reduced concentrations of Ile and Gln, hypoxanthine was added to 3 μM, heat-inactivated human serum extensively dialysed against water was added to 10%, and 2x amino acid free media was added to a final concentration of 1x. Infected RBCs were washed in 1x RPMI without amino acids, then in the appropriate media, before growing in the presence of inhibitors for 72 h as in the parasite growth assay listed above. Parasite growth was determined by *Pf*LDH assay.

## Additional Information

**How to cite this article**: Dickerman, B. K. *et al*. Identification of inhibitors that dually target the new permeability pathway and dihydroorotate dehydrogenase in the blood stage of *Plasmodium falciparum*. *Sci. Rep*. **6**, 37502; doi: 10.1038/srep37502 (2016).

**Publisher's note:** Springer Nature remains neutral with regard to jurisdictional claims in published maps and institutional affiliations.

## Supplementary Material

Supplementary Information

Supplementary Table S1

Supplementary Table S2

## Figures and Tables

**Table 1:** EC_50_ values for inhibition of sorbitol-mediated lysis (NPP activity) and growth of *P. falciparum* blood stage parasites treated with the compounds indicated.

	EC_50_ for Sorbitol Lysis	EC_50_ for Parasite Growth
NPPB	**1.23 μM**	**62.6 μM**
	(*1.1–1.4*)	(*49–80*)
Furosemide	**11.8 μM**	**134 μM**
	(*10–14*)	(*120–150*)
MMV020439	**222 nM**	**329 nM**
	(*170–280*)	(*280–390*)
MMV007571	**367 nM**	**857 nM**
	(*310–440*)	(*720–1000*)

The EC_50_ values (in bold) were calculated from the pooled data from three independent replicates of three technical replicates each. 95% confidence intervals are listed in parenthesis.

**Table 2:** The fold change in the EC_50_ of parasite growth following drug treatment between 3D7 parasites and two independently transfected lines expressing yeast DHODH (yDHODH).

	EC_50_ for Parasite Growth			Fold Increase	
	3D7	yDHODH #1	yDHODH #2	yDHODH #1	yDHODH #2
ATV	**176** **pM** (*130*–*230*)	**>1.25 μM**	**>1.25 μM**	>7100	>7100
DSM-1	**64.8 nM** (*55*–*77*)	**>6.25 μM**	**>6.25 μM**	>96	>96
NPPB	**54.2 μM** (*43*–*68*)	**58.6 μM** (*48*–*72*)	**60.5 μM** (*53*–*69*)	1.1 (*p =0.6360*)	1.1 (p **=** *0.4643*)
Furosemide	**116 μM** (*97*–*140*)	**127 μM** (*100*–*160*)	**107 μM** (*89*–*130*)	1.1 (*p =0.5706*)	0.9 (p = *0.5556*)
MMV020439	**278 nM** (*230*–*340*)	**1.79 μM** (*1.5*–*2.2*)	**1.66 μM** (*1.3*–*2.1*)	**6.4 (*p =0.0002*)**	**6.0 (p = *0.0003*)**
MMV007571	**1.04 μM** (*0.92*–*1.2*)	35.4 μM* (*24*–*63*)	40.2 μM * (*26*–*87*)	**34 (*p =0.0031*)**	**39 (p = *0.0054*)**

The EC_50_ values (in bold) were calculated from the pooled data from three independent replicates of three technical replicates each. 95% confidence intervals are listed in parenthesis. *Denotes values extrapolated from regression where inhibition did not reach 50%. P-values for fold change (comparing EC_50_ from 3D7 with corresponding yDHODH) were calculated using a two-tailed Student’s t-test with Welch’s correction, p-values in bold signify <0.05.

**Table 3:** The change in the EC_50_ of parasite growth following drug treatment between 3D7 parasites and those expressing yeast DHODH (yDHODH) in the presence of proguanil.

	Control		+ 1 μM Proguanil	
	3D7	yDHODH	3D7	yDHODH
ATV	**370 pM** (*290*–*480*)	**>2.5 μM**	**33.4 pM** (*25*–*45*)	**42.3 pM** (*31*–*59*)
DSM-1	**56.5 nM** (*46*–*70*)	**>2.5 μM**	**78.7 nM** (*62*–*100*)	**1.53 μM** (*120*–*210*)
MMV020439	**288 nM** (*230*–*370*)	**2.93 μM** (*2.2 to 4.0*)	**20.2 nM** (*16*–*26*)	**30.8 nM** (*23*–*42*)
MMV007571	**1.02 μM** (*0.84*–*1.2*)	97.5 μM* (*55*–*230*)	**291 nM** (*240*–*360*)	**436 nM** (*340*–*560*)

The EC_50_ values (in bold) were calculated from the pooled data from three independent replicates of three technical replicates each. 95% confidence intervals are listed in parenthesis. *Denotes value extrapolated from regression where inhibition did not reach 50%.

**Table 4:** The fold change in the EC_50_ of parasite growth following drug treatment between 3D7 parasites and those expressing yeast DHODH (yDHODH) in the presence of proguanil (from Table 3), and the fold change in 3D7 parasites treated with each drug in the presence and absence of proguanil.

	Fold Increase with yDHODH		3D7 Fold Decrease with Proguanil
	Control	+1 μM Proguanil	
ATV	>50	1.3 (p = *0.3158*)	**11 (*p =0.0002*)**
DSM-1	>44	**19 (p = *0.0001*)**	0.7 ( *p =0.1105*)
MMV020439	**10 (*p = 0.0004*)**	1.5 (p = *0.1088*)	**14 (*p =0.0001*)**
MMV007571	**96 (*p = 0.0054*)**	1.5 (p = *0.0709*)	**3.5 (*p =0.001*)**

P-values for fold change (comparing EC_50_ from 3D7 with corresponding yDHODH and comparing 3D7 with and without proguanil) were calculated using a two-tailed Student’s t-test with Welch’s correction, p-values in bold signify <0.05.

**Table 5:** Comparison of the EC_50_ of parasite growth and IC_50_ for direct inhibition of *in vitro Pf*DHODH activity.

	EC_50_ for Parasite Growth Inhibition	IC_50_ for *in vitro* DHODH Inhibition
ATV	**176 pM** (*130*–*230*)	**>50 μM**
DSM-1	**64.8 nM** (*55*–*77*)	**441 nM** (*130*–*1500*)
MMV020439	**278 nM** (*230*–*340*)	**>250 μM**
MMV007571	**1.04 μM** (*0.92*–*1.2*)	158 μM* (*54*–*1200*)

The EC_50_ values are taken from Table 1, IC_50_ values were calculated from pooled data from three independent replicates of two technical replicates each. 95% confidence intervals are listed in parenthesis. *Denotes values extrapolated from regression where inhibition did not reach 50%.

**Table 6:** The EC_50_ of parasite growth following drug treatment of 3D7 and yDHODH expressing parasites in normal media and reduced isoleucine, glutamine and hypoxanthine media (PGIM).

	Normal Media		PGIM Media (Low AA)	
	3D7	yDHODH	3D7	yDHODH
NPPB	**65.8 μM** (54–*)	**87 μM** (67–*)	**41.6 μM** (32–55)	**53.4 μM** (36–86)
Furosemide	**238 μM** (170–330)	**265 μM** (200–350)	**73.1 μM** (43–130)	**117 μM** (76–190)
MMV020439	**223 μM** (100–510)	**11.5 μM** (7.1–20)	**39.3 μM** (19–77)	**169 μM** (59–490)
MMV007571	**2.48 μM** (1.5–4.2)	**41.3 μM** (29–64)	**1.67 μM** (1.2–2.3)	**11.6 μM** (5.2–32)

The EC_50_ values (in bold) were calculated from the pooled data from three independent replicates of two technical replicates each. 95% confidence intervals are listed in parenthesis. *Denotes the upper range was not resolved.

**Table 7:** The fold change in the EC_50_ of parasite growth following drug treatment of 3D7 parasites in normal and PGIM media, and fold change between 3D7 and yDHODH expressing parasites in normal and PGIM media.

	3D7 EC_50_ Fold Decrease with PGIM	EC_50_ Fold Increase with yDHODH	
		Normal	PGIM
NPPB	1.6 (*p* = *0.0523*)	1.3(*p* = *0.5614*)	1.3 (*p *=* 0.3463)*
Furosemide	**3.3 (*****p *****= *****0.0216***)	1.1 (*p* = *0.6417*)	1.6 (*p* = *0.2263*)
MMV020439	**5.7 (*****p = 0.0304***)	**52 (*****p***** = *****0.0023***)	4.3 (*p* = *0.0851*)
MMV007571	1.5 (*p* = *0.2425*)	**17 (*****p***** = *****0.001***)	**6.9 (*****p***** = *****0.0321***)

P-values were calculated by two-tailed Student’s t-test with Welch’s correction, numbers in bold signify a p-value < 0.05.
