# Supplementary material for: Identification of inhibitors that dually target the new permeability pathway and dihydroorotate dehydrogenase in the blood stage of Plasmodium falciparum
Source: Sci Rep. 2016 Nov 22;6:37502. doi: 10.1038/srep37502 (PMC5118696; doi:10.1038/srep37502)
Supplement: Supplementary Information [file srep37502-s1.pdf]

Suuplemental Figures and Figure Legends for:

Identification of inhibitors that dually target the new permeability pathway and dihydroorotate dehydrogenase in the blood stage of *Plasmodium falciparum*.

Benjamin K. Dickerman[1†], Brendan Elsworth[1,3†], Simon A. Cobbold[2], Catherine Q. Nie[1], Malcolm J. McConville[2], Brendan S. Crabb[1,2,3] and Paul R. Gilson[1,3]*

1. Burnet Institute, Melbourne, Victoria, Australia

2. Bio21 Institute of Molecular Science and Biotechnology, University of Melbourne, Parkville, Victoria, Australia

3. Monash University, Clayton, Victoria, Australia

† These authors contributed equally

*Correspondence to paul.gilson@burnet.edu.au

Figure S1

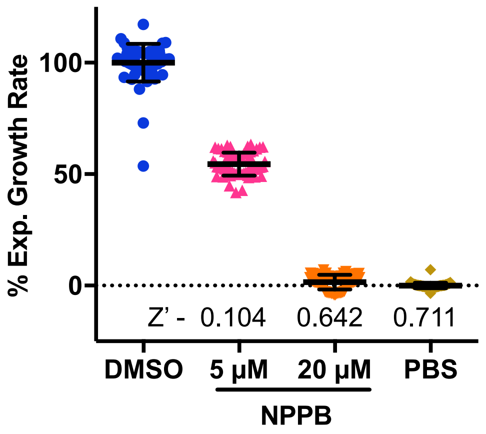

% Exp. Growth Rate
100
50
0
Z' - 0.104 0.642 0.711
DMSO
5 µM
20 µM
PBS
NPPB

Figure S2

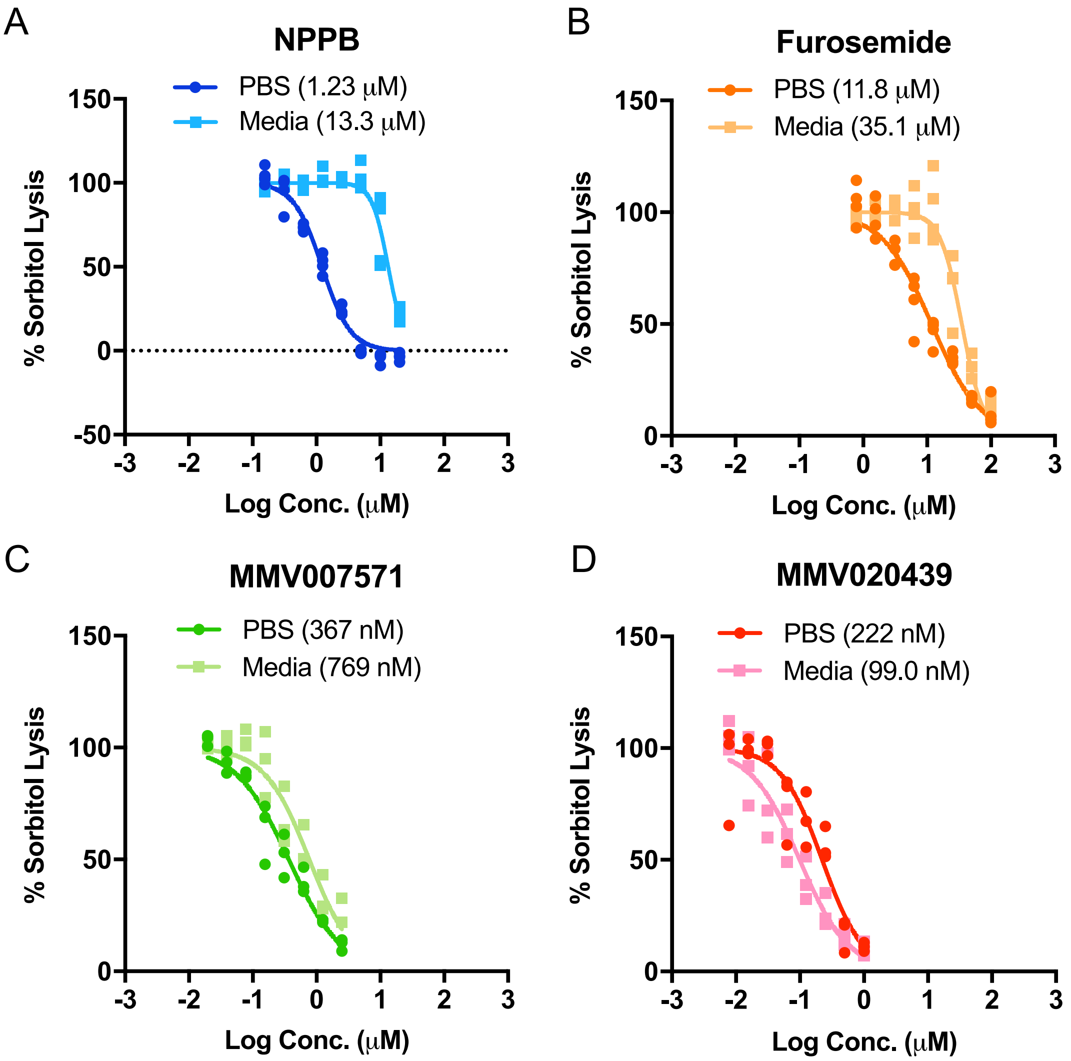
A
NPPB
PBS (1.23 μM)
Media (13.3 μM)
% Sorbitol Lysis
Log Conc. (μM)
B
Furosemide
PBS (11.8 μM)
Media (35.1 μM)
% Sorbitol Lysis
Log Conc. (μM)
C
MMV007571
PBS (367 nM)
Media (769 nM)
% Sorbitol Lysis
Log Conc. (μM)
D
MMV020439
PBS (222 nM)
Media (99.0 nM)
% Sorbitol Lysis
Log Conc. (μM)

Figure S3

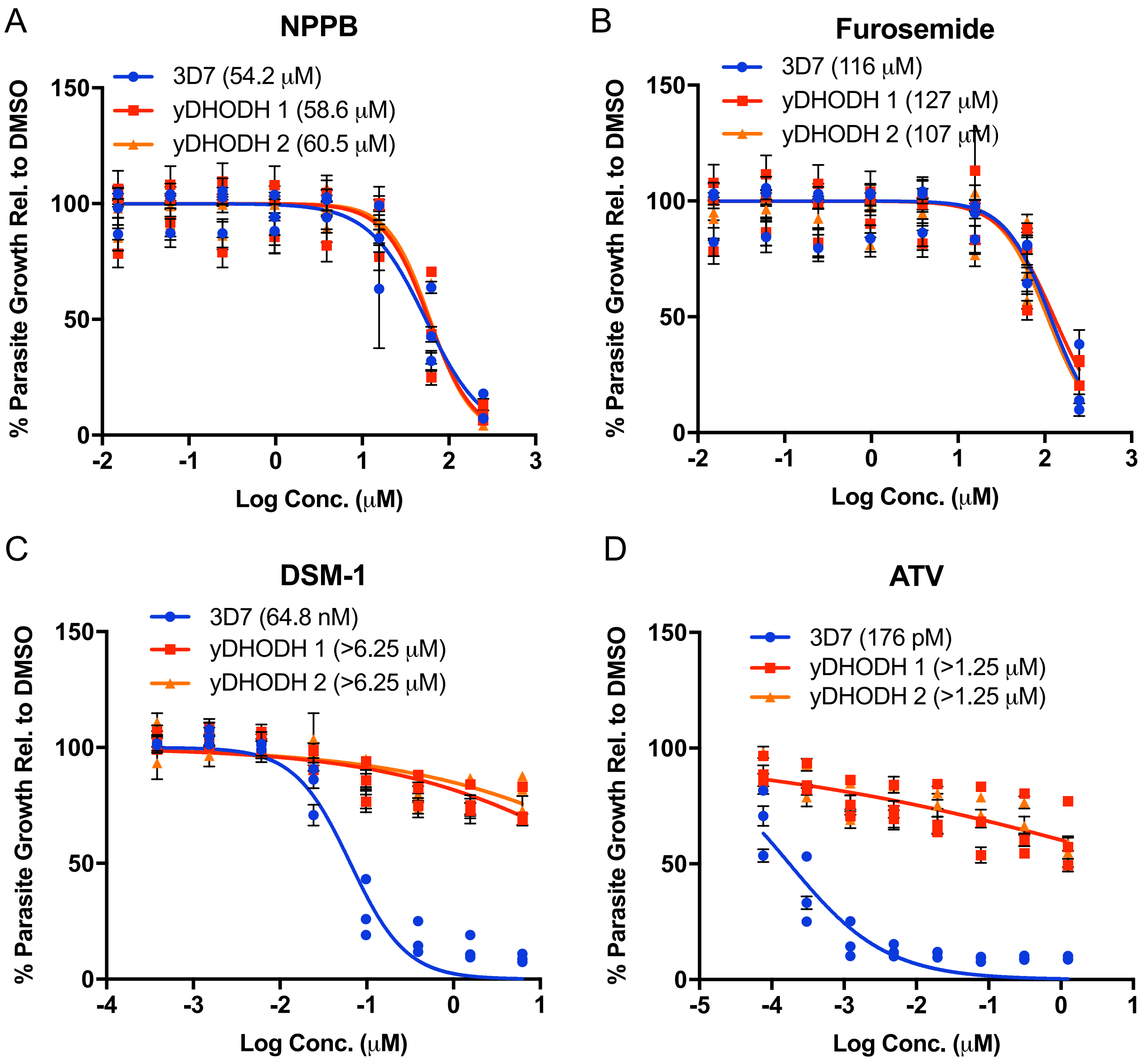
A
NPPB
3D7 (54.2 μM)
yDHODH 1 (58.6 μM)
yDHODH 2 (60.5 μM)
% Parasite Growth Rel. to DMSO
Log Conc. (μM)
B
Furosemide
3D7 (116 μM)
yDHODH 1 (127 μM)
yDHODH 2 (107 μM)
% Parasite Growth Rel. to DMSO
Log Conc. (μM)
C
DSM-1
3D7 (64.8 nM)
yDHODH 1 (>6.25 μM)
yDHODH 2 (>6.25 μM)
% Parasite Growth Rel. to DMSO
Log Conc. (μM)
D
ATV
3D7 (176 pM)
yDHODH 1 (>1.25 μM)
yDHODH 2 (>1.25 μM)
% Parasite Growth Rel. to DMSO
Log Conc. (μM)

Figure S4

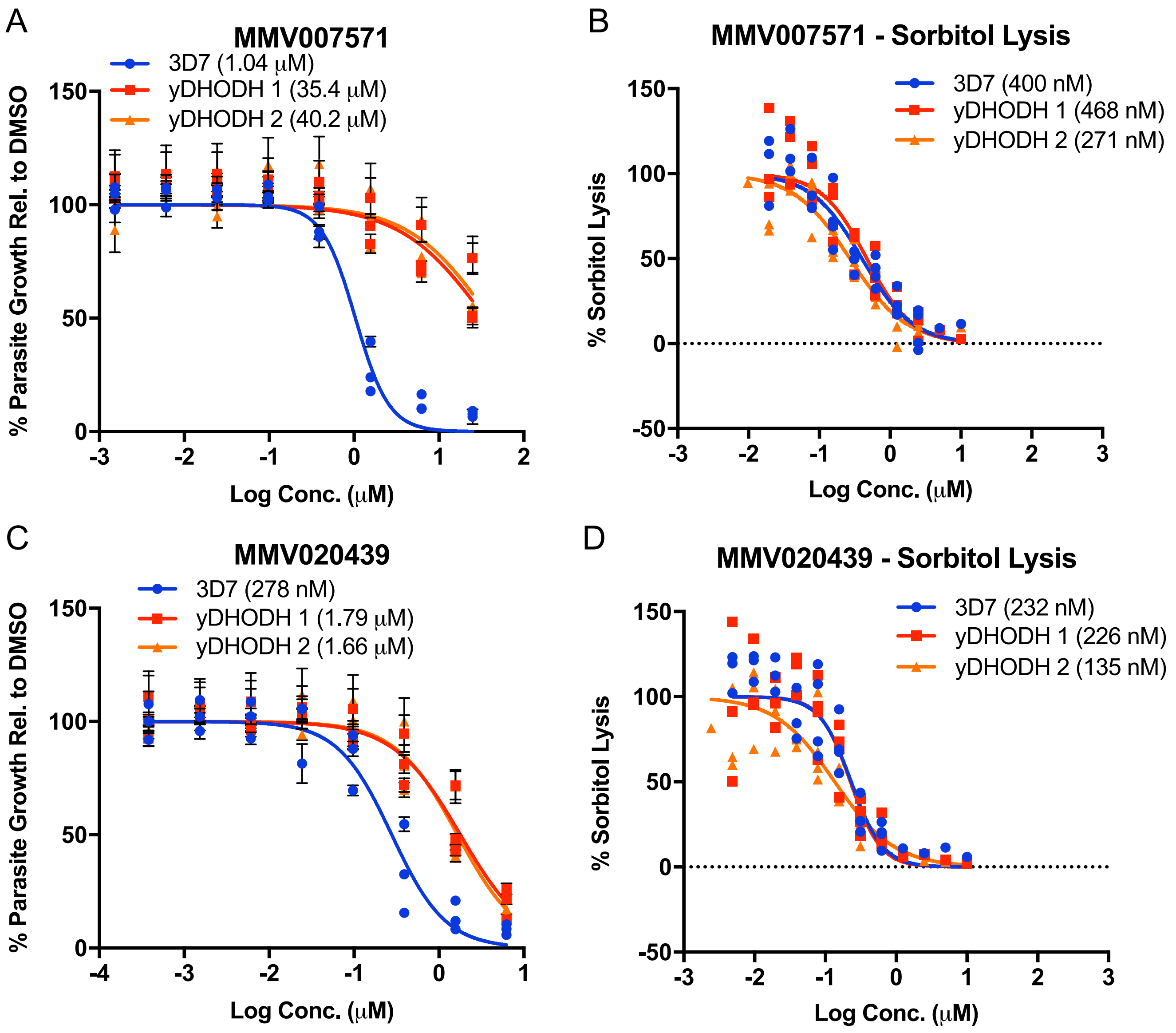
A
MMV007571
3D7 (1.04 µM)
yDHODH 1 (35.4 µM)
yDHODH 2 (40.2 µM)
% Parasite Growth Rel. to DMSO
Log Conc. (µM)
B
MMV007571 - Sorbitol Lysis
3D7 (400 nM)
yDHODH 1 (468 nM)
yDHODH 2 (271 nM)
% Sorbitol Lysis
Log Conc. (µM)
C
MMV020439
3D7 (278 nM)
yDHODH 1 (1.79 µM)
yDHODH 2 (1.66 µM)
% Parasite Growth Rel. to DMSO
Log Conc. (µM)
D
MMV020439 - Sorbitol Lysis
3D7 (232 nM)
yDHODH 1 (226 nM)
yDHODH 2 (135 nM)
% Sorbitol Lysis
Log Conc. (µM)

Figure S5

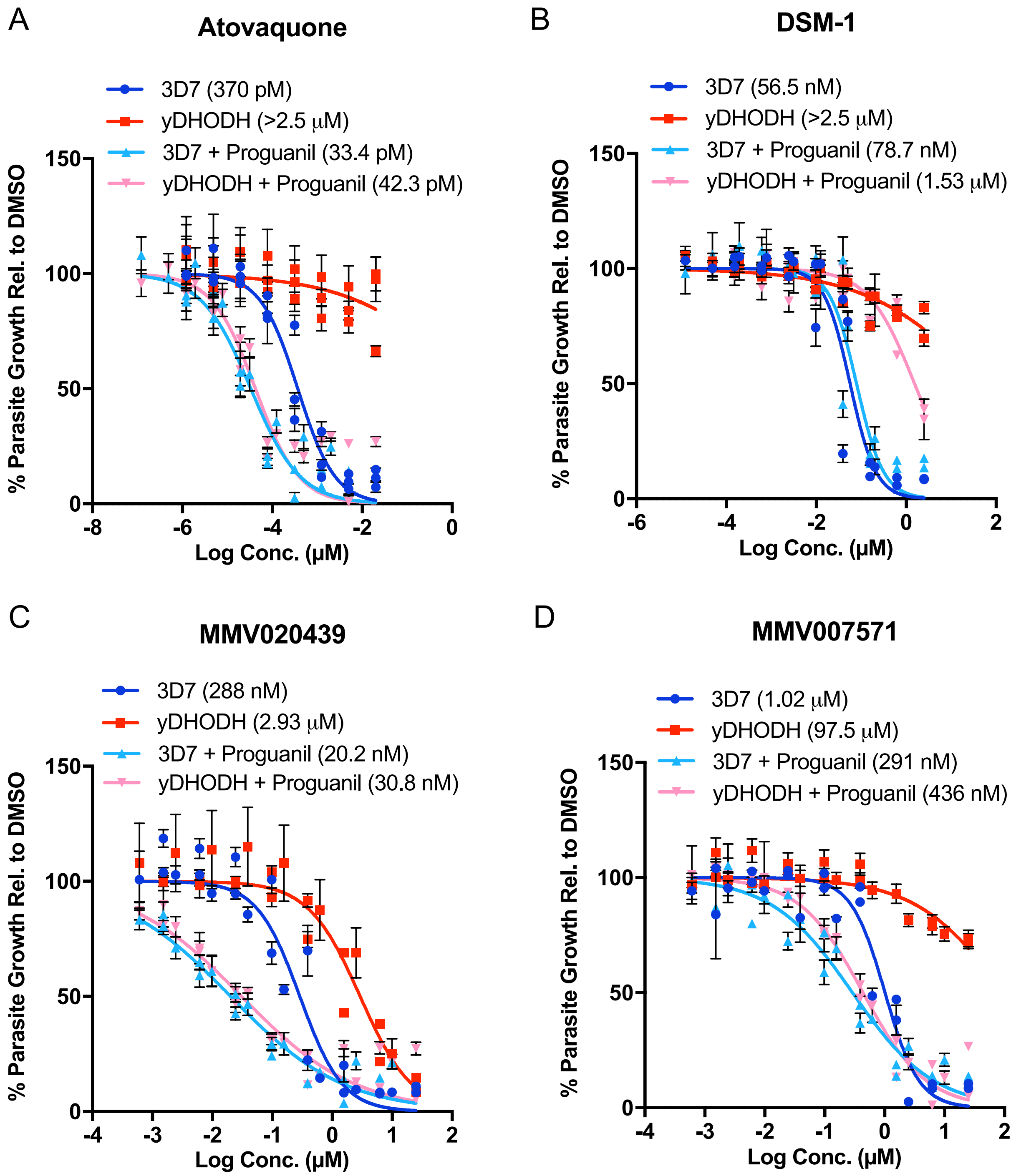
A
Atovaquone
3D7 (370 pM)
yDHODH (>2.5 μM)
3D7 + Proguanil (33.4 pM)
yDHODH + Proguanil (42.3 pM)
% Parasite Growth Rel. to DMSO
Log Conc. (μM)
150
100
50
0
-8
-6
-4
-2
0
B
DSM-1
3D7 (56.5 nM)
yDHODH (>2.5 μM)
3D7 + Proguanil (78.7 nM)
yDHODH + Proguanil (1.53 μM)
% Parasite Growth Rel. to DMSO
Log Conc. (μM)
150
100
50
0
-6
-4
-2
0
2
C
MMV020439
3D7 (288 nM)
yDHODH (2.93 μM)
3D7 + Proguanil (20.2 nM)
yDHODH + Proguanil (30.8 nM)
% Parasite Growth Rel. to DMSO
Log Conc. (μM)
150
100
50
0
-4
-3
-2
-1
0
1
2
D
MMV007571
3D7 (1.02 μM)
yDHODH (97.5 μM)
3D7 + Proguanil (291 nM)
yDHODH + Proguanil (436 nM)
% Parasite Growth Rel. to DMSO
Log Conc. (μM)
150
100
50
0
-4
-3
-2
-1
0
1
2

Figure S6

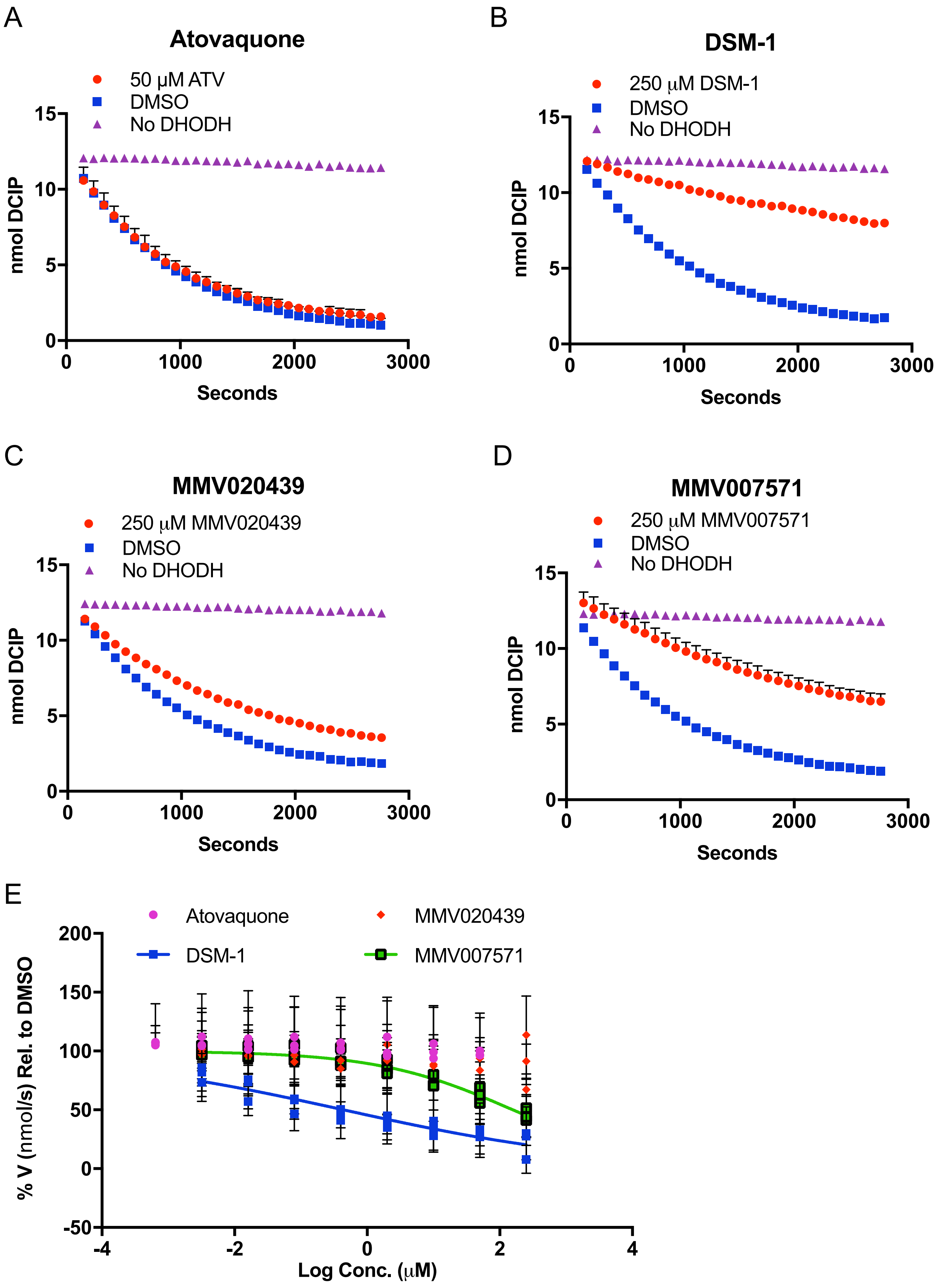
A
Atovaquone
50 µM ATV
DMSO
No DHODH
nmol DCIP
Seconds
B
DSM-1
250 µM DSM-1
DMSO
No DHODH
nmol DCIP
Seconds
C
MMV020439
250 µM MMV020439
DMSO
No DHODH
nmol DCIP
Seconds
D
MMV007571
250 µM MMV007571
DMSO
No DHODH
nmol DCIP
Seconds
E
Atovaquone
MMV020439
DSM-1
MMV007571
% V (nmol/s) Rel. to DMSO
Log Conc. (µM)

Figure S7

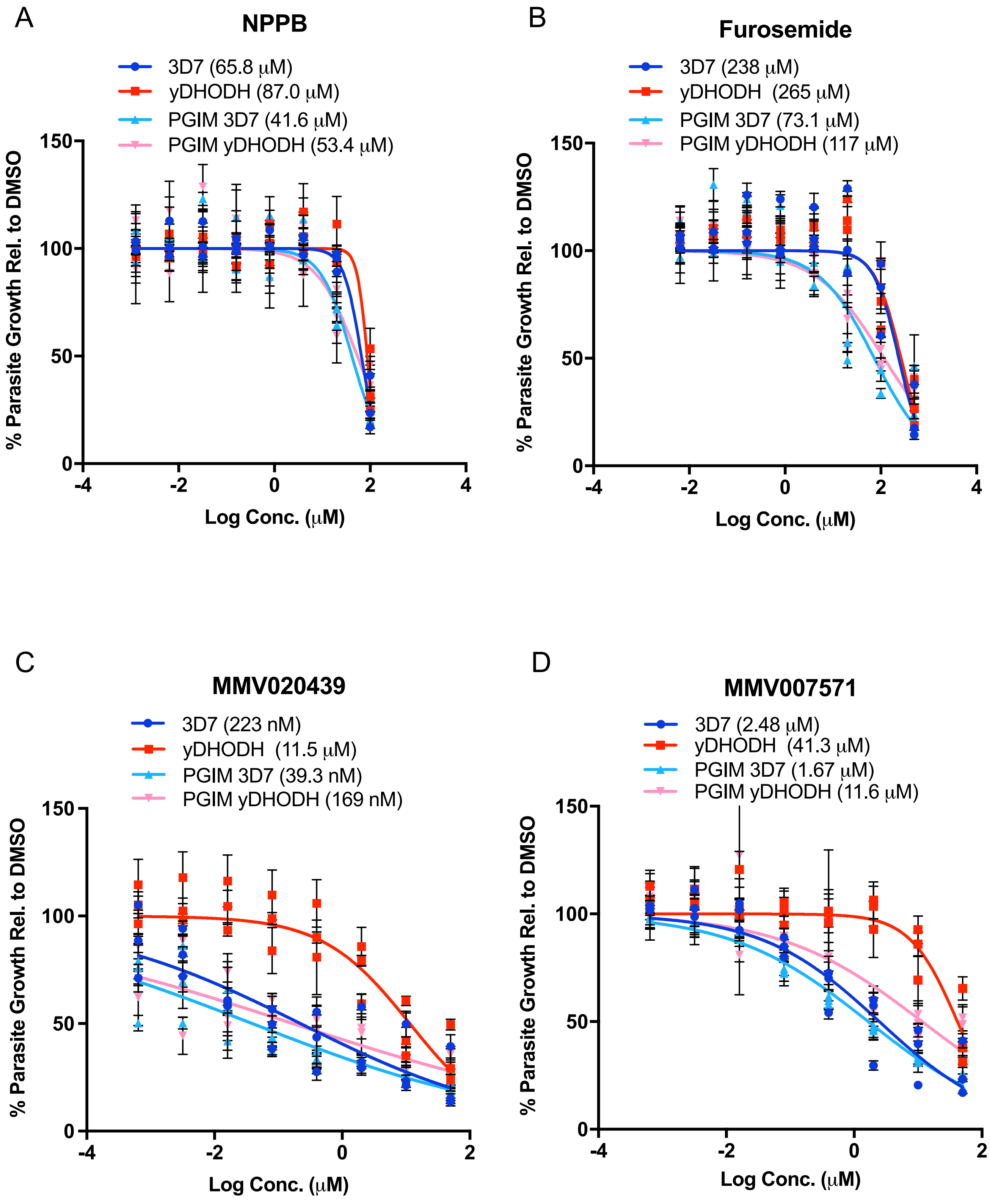
A
NPPB
3D7 (65.8 μM)
yDHODH (87.0 μM)
PGIM 3D7 (41.6 μM)
PGIM yDHODH (53.4 μM)
% Parasite Growth Rel. to DMSO
150
100
50
0
-4
-2
0
2
4
Log Conc. (μM)
B
Furosemide
3D7 (238 μM)
yDHODH (265 μM)
PGIM 3D7 (73.1 μM)
PGIM yDHODH (117 μM)
% Parasite Growth Rel. to DMSO
Log Conc. (μM)
C
MMV020439
3D7 (223 nM)
yDHODH (11.5 μM)
PGIM 3D7 (39.3 nM)
PGIM yDHODH (169 nM)
% Parasite Growth Rel. to DMSO
Log Conc. (μM)
D
MMV007571
3D7 (2.48 μM)
yDHODH (41.3 μM)
PGIM 3D7 (1.67 μM)
PGIM yDHODH (11.6 μM)
% Parasite Growth Rel. to DMSO
Log Conc. (μM)

Supplementary Figure Legends

Supplemental Figure 1. Reanalysis of NPPB validation data using exponential growth rate in place of individual timepoints.

Supplemental Figure 2. The EC_{50} for sorbitiol lysis in PBS buffer compared with serum-containing culture media. Each point represents the lysis rate calculated from three technical replicates. Data from three (MMV007571 and MMV020439) or four (NPPB and Furosemide) independent replicates were pooled to calculate EC_{50}.

Supplemental Figure 3. The growth of yDHODH-expressing parasites is less susceptible to the *Pf*DHODH inhibitors atovaquone and DSM-1, but not NPP inhibitors NPPB and furosemide. > 2.5 µM and >6.25 µM indicate EC_{50} not determined, and the maximum concentration tested. Each point represents the % Absorbance (620nm) relative to DMSO control +/- SEM from three technical replicates, data from three independent experiments were pooled to calculate EC_{50}.

Supplemental Figure 4. Expression of yDHODH in Nanoluciferase (NLuc) expressing *P. falciparum* only partially reverses the effects of MMV007571 and MMV020439 upon parasite proliferation. The EC_{50} of NLuc expressing 3D7 parasites +/- yDHODH, for parasite growth (**A, C**) and sorbitol lysis (**B, D**) were determined by NLuc activity and *Pf*LDH assays. Each point in A&C represents the % Absorbance (620nm) relative to DMSO control +/- SEM from three technical replicates, each point in B&D represents the lysis rate calculated from three technical replicates. Data from three independent experiments were pooled to calculate

EC_{50}.Values in parentheses represent EC_{50}. Data from this figure are summarised in Table 2.

Supplemental Figure 5. Treatment of 3D7 and yDHODH expressing parasites with MMV007571 or MMV020439 together with Proguanil . The EC_{50} for growth of 3D7 parasites with or without yDHODH, when treated with or without 1 μM proguanil together with atovaquone (A), DSM-1 (B), MMV020439 (C) or MMV007571 (D) was determined by *Pf*LDH assay. Each point represents the % Absorbance (620nm) relative to DMSO control +/- SEM from three technical replicates, data from three independent experiments were pooled to calculate EC_{50}. Values in parentheses represent EC_{50}. Data from this figure are summarised in Tables 3&4.

Supplemental Figure 6. *in vitro* DHODH activity with MMV007571 or MMV020439. Recombinant DHODH activity was monitored by measuring absorbance at 620nm to detect DCIP reduction and plotting against time (s) to calculate enzyme velocity (nmol/s). Representative data with the highest concentration tested of Atovaquone (A), DSM-1 (B), MMV020439 (C) or MMV007571 (D) is shown. Each point in A-D represents nmol DCIP as calculated from a standard curve +/- SEM from two technical replicates. IC_{50} was calculated by plotting enzyme velocity (relative to DMSO control) against inhibitor concentration (E). Each point represents the % enzyme velocity (nmol/s) rel to DMSO +/- SEM from two technical replaicates, data from three independent experiments were pooled to calculate IC_{50}. Data from this figure are summarised in Table 5.

Supplemental Figure 7. Treatment of 3D7 and yDHODH expressing parasites with MMV007571 or MMV020439 in reduced nutrient media . The EC_{50} for growth of 3D7 parasites with or without yDHODH for atovaquone (A), DSM-1 (B), MMV020439 (C) or MMV007571 (D) was determined by *Pf*LDH assay in normal media or media with reduced concentrations of Ile, Gln and hypoxanthine (PGIM). Each point represents the % Absorbance (620nm) relative to DMSO control +/- SEM from three technical replicates, data from three independent experiments were pooled to calculate EC_{50}. Values in parentheses represent EC_{50}. Data from this figure are summarised in Tables 6&7.
